# Random Forest-Based Approach for Maximum Power Point Tracking of Photovoltaic Systems Operating under Actual Environmental Conditions

**DOI:** 10.1155/2017/1673864

**Published:** 2017-06-15

**Authors:** Hussain Shareef, Ammar Hussein Mutlag, Azah Mohamed

**Affiliations:** ^1^Department of Electrical Engineering, College of Engineering, United Arab Emirates University, P.O. Box 15551, Al Ain, UAE; ^2^Department of Computer Engineering Techniques, Electrical Engineering Technical College, Middle Technical University, Baghdad, Iraq; ^3^Department of Electrical, Electronic and Systems Engineering, Faculty of Engineering and Built Environment, Universiti Kebangsaan Malaysia, 43600 Bangi, Selangor, Malaysia

## Abstract

Many maximum power point tracking (MPPT) algorithms have been developed in recent years to maximize the produced PV energy. These algorithms are not sufficiently robust because of fast-changing environmental conditions, efficiency, accuracy at steady-state value, and dynamics of the tracking algorithm. Thus, this paper proposes a new random forest (RF) model to improve MPPT performance. The RF model has the ability to capture the nonlinear association of patterns between predictors, such as irradiance and temperature, to determine accurate maximum power point. A RF-based tracker is designed for 25 SolarTIFSTF-120P6 PV modules, with the capacity of 3 kW peak using two high-speed sensors. For this purpose, a complete PV system is modeled using 300,000 data samples and simulated using the MATLAB/SIMULINK package. The proposed RF-based MPPT is then tested under actual environmental conditions for 24 days to validate the accuracy and dynamic response. The response of the RF-based MPPT model is also compared with that of the artificial neural network and adaptive neurofuzzy inference system algorithms for further validation. The results show that the proposed MPPT technique gives significant improvement compared with that of other techniques. In addition, the RF model passes the Bland–Altman test, with more than 95 percent acceptability.

## 1. Introduction

Solar energy is inexhaustible, free, and clean and is considered as the core of renewable energy (RE) in recent times primarily because of the depletion of fossil fuels and environmental pollution [1]. Among various RE resources, photovoltaic (PV) systems are gaining popularity in a wide range of applications, from small building integrated systems to large-scale utility systems [2]. However, PV systems have the issue of intermittent power generation under different weather conditions [3]. Moreover, the amount of generated power from a solar cell depends on the nonlinear power-voltage (*P*-*V*) and current-voltage (*I*-*V*) characteristics that vary with irradiance (*G*) and temperature (*T*) [4]. Regardless of the size and type, the crucial issue for any PV system is the efficiency of the algorithm used to track the maximum power point (MPP). Thus, interest in improving maximum power point tracking (MPPT) algorithms is gaining its momentum among PV research communities [5]. The MPPT is a unique point on the* P*-*V* curves, where maximum power is provided [6]. Many MPPT methods were proposed in the literature since the 1960s. These methods can be grouped into two types, namely, conventional MPPT approaches and soft computing-based MPPT approaches.

Among conventional approaches, the most dominant methods are Incremental Conductance (IC) [7], Hill Climbing (HC) [8, 9], and Perturb and Observe (P&O) [10, 11] methods. The P&O method presents a perturbation (*Ф*) in the operating current and voltage of a PV system and then observes the change in power in the system. The idea is to observe whether the converter power is increasing toward the MPP and in the next step, while the reference current/voltage is increased by the amount of *Ф*. The P&O method depends on the applied step size for the current/voltage reference. However, oscillations occur around the MPP, which leads to power loss. To avoid large oscillations, [12] suggested minimizing the applied step compromising the response time of the method. Meanwhile, the HC technique is highly comparable with P&O. The difference between P&O and HC methods is that the latter updates the operating point for the PV system by perturbing the duty cycle instead of the current/voltage. If the direction of the power is increasing, updating at the operating point is achieved by perturbing the duty cycle through the applied step size. Otherwise, the tracking is indicated as moving away from the MPP. However, HC is prone to failure in cases of large changes in irradiance [13]. To overcome some of the limitations of the P&O and HC methods, the IC approach was proposed under the conventional MPPT category. The idea behind the IC operation is to determine the MPP by tracking the PV panel power against the voltage curve [14]. This method improves dynamic performance and tracking accuracy under rapidly changing environment conditions. However, the IC method also suffers from some oscillation around the MPP, aside from power losses caused by noise and measurement errors. Furthermore, the IC method has higher computational burdens than the P&O method.

In the soft computing-based MPPT category, the most talked about approaches are Fuzzy Logic Control (FLC) [15], artificial neural network (ANN) [16], and other Computational Intelligence (CI) [17] methods. The main advantage of FLC-based methods is that a mathematical model for the system is not required. Thus, the FLC-based MPPT has been frequently implemented with PV systems in recent years [18, 19]. However, the performance of FLC depends on the rule basis, number of rules, and membership function [20]. These variables are determined by a trial and error procedure, which is time-consuming. Another well-known approach in this category is ANN. In the MPPT application, ANN is applied to estimate and recognize unknown parameters [21] such as reference current/voltage or duty cycles. However, weights associated with the neurons should be accurately determined by a training process before they are used to supply the reference current (*I*_MPP_) or reference voltage (*V*_MPP_) to the MPPT controller. Besides, the ANN requires large training data before the method can be trained and implemented in the MPPT system. Another popular soft computing method for MPPT is based on CI methods which are nature-inspired computational methodologies that address complex real-world problems. These methods can be divided into two groups: swarm intelligence algorithms (SAs) and evolutionary algorithms (EAs). The most popular SAs are particle swarm optimization (PSO) [22], artificial bee colony (ABC) [23], and ant colony optimization (ACO) [24]. The most popular EAs are the genetic algorithm (GA) [25], differential evolution [26], and lightning search algorithm [27]. PSO has been used to optimize a nine-rule FLC for MPPT in a grid-connected PV inverter in which the FLC generates a DC bus voltage reference for MPPT [28]. A hybrid GA-ANN MPPT is proposed in [29]. In this approach, the optimized values for the array voltage and power are obtained by GA for different irradiance and temperature conditions. Similarly, the authors in [30] used GA to optimize the FLC-based MPPT. However, CI methods have limiting factors such as trapping in local minima and premature convergence. Among the aforementioned methods, most have been criticized for being inefficient because of the inability of the detector to fully differentiate the accurate MPP. Current challenges in detecting accurate MPP lie in the adaptation of algorithms in fast-changing environmental conditions, efficiency, accuracy at steady state, and the response speed of the tracking algorithm. In a number of previous studies, actual environmental condition problems were not addressed fully. Hence, the aforementioned methods do not have an integrated solution to address all of the problems in real environment conditions and are therefore inadequate in producing an effective MPPT system.

Recently, a new soft computing approach known as random forest (RF) approach received attention in many applications. The authors in [31] present a supervised classification method based on the RF to identify the layer from where groundwater samples were extracted, and they reported that the results by the RF approach were much better than those by linear discriminant analysis and decision tree-supervised classification methods. Ash Booth et al. in [32] proposed an expert system that uses novel RF machine learning techniques to predict the price return over seasonal events, and then these predictions are used to develop a profitable trading strategy. The results show that the RF approach produces superior results in terms of both profitability and prediction accuracy compared with those of other ensemble techniques. The RF method was also applied in other applications such as in improving rainfall rate assignment [33], assessing visual attention [34], resampling field spectra [35], and quantification of aboveground biomass [36]. In these applications, the authors concluded that the RF model has higher stability and robustness and better success rates with the use of proper training parameters than those of other models. Therefore, a better outcome will be obtained with the implementation of the RF approach in MPPT for PV systems.

This paper attempts to design and implement the RF method to track MPP accurately for the PV system, by considering the problems of the fast-changing environmental conditions. The system is modeled in the MATLAB environment to demonstrate the performance of the proposed controller.

## 2. PV Model and Maximum Power Point

**Figure 1:**
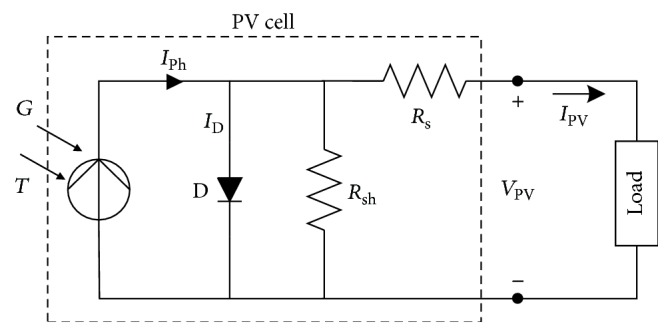
Electrical equivalent circuit of PV cell.

The power output of the PV system depends on its voltage and current characteristics. However, solar irradiation and temperature are the two main parameters responsible for the operating point of the PV panel, hence, the MPP [37]. The equivalent electrical circuit for the PV is shown in Figure 1, which is used to obtain the characteristics of a PV cell. The electrical circuit contains a diode, a serial resistor, a parallel-connected resistor, and a current source. The mathematical model of the circuit, which represents the output of the cell current *I*, can be expressed as follows [38]:

(1) $$\begin{array}{c} I_{PV}=I_{ph}-I_{o}\left(\;e^{\left(q\left(V+I\cdot R_{s}\right)/\left(n\cdot K_{B}\cdot T\right)\right)}-1\;\right)-\frac{V+I\cdot R_{s}}{R_{sh}}, \end{array}$$

where *I*_PV_ is cell output current (A), *I*_ph_ is the light-generated current (A), *I*_*o*_ is the cell reverse saturation current or dark current (A), *q* is the electronic charge (1.6*∗*10^−19^ C),* V* is the cell output voltage (V), *n* is the ideality factor, *K*_*B*_ is the Boltzmann's constant (1.38*∗*10^−23^ J/K), and* T* is the cell temperature (K).

The light-generated current extracted from the photovoltaic cell, *I*_ph_, is directly proportional to the solar irradiance, *G*, and temperature,* T*. Assuming the nominal condition for *G* and *T* denoted by *G*_*n*_ and *T*_*n*_, respectively, *I*_ph_ at other conditions can be calculated as follows [38]:

(2) $$\begin{array}{c} I_{ph}=\left[\;I_{sc,n}+\alpha \left(\;T-T_{n}\;\right)\;\right]\frac{G}{G_{n}}, \end{array}$$

where *I*_sc,*n*_ is short-circuit current at the nominal condition and *α* is short-circuit current temperature coefficient which are provided by the manufacture's datasheet as shown in Table 1.

**Figure 2:**
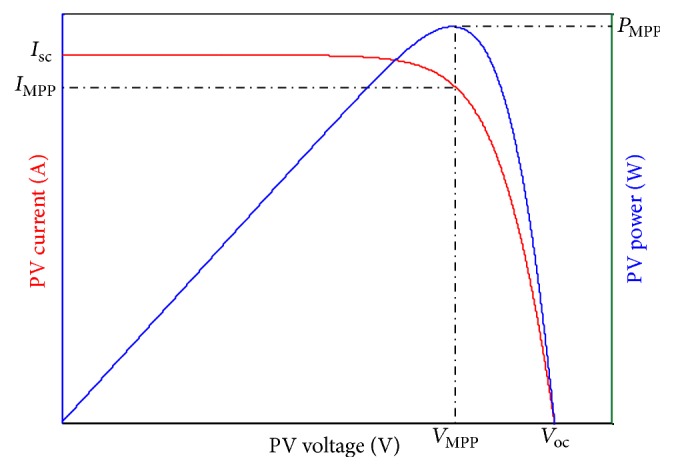
Current-voltage (*I*-*V*) and power-voltage (*P*-*V*) characteristic curves of a solar cell.

Since electric power is the product of current and voltage, therefore a power-voltage (*P-V*) characteristic curve of a solar cell can be obtained for a given radiation level as shown in Figure 2. From the figure, at the maximum short-circuit current, the voltage is zero and thus the power is also zero. The situation for current and voltage is reversed at the open-circuit point, so again the power here is zero. However, there is one particular point at which the solar cell can deliver maximum power for a given radiation intensity, and this operating point is called the maximum power point (MPP) point. From (1) and (2), the cell output current is shown to be nonlinear and dependent on irradiation and temperature. These equations can be used to calculate reference current (*I*_MPP_) which eventually provides MPP by considering the cell output voltage. If the number of PV cells is known, the same relationship can be used to obtain MPP in a PV module or a system. However, the main drawback of this mathematical model is the time-consuming and iterative process required to calculate the cell output current, which hinders the utilization of the model in high-speed tracking.

**Figure 3:**
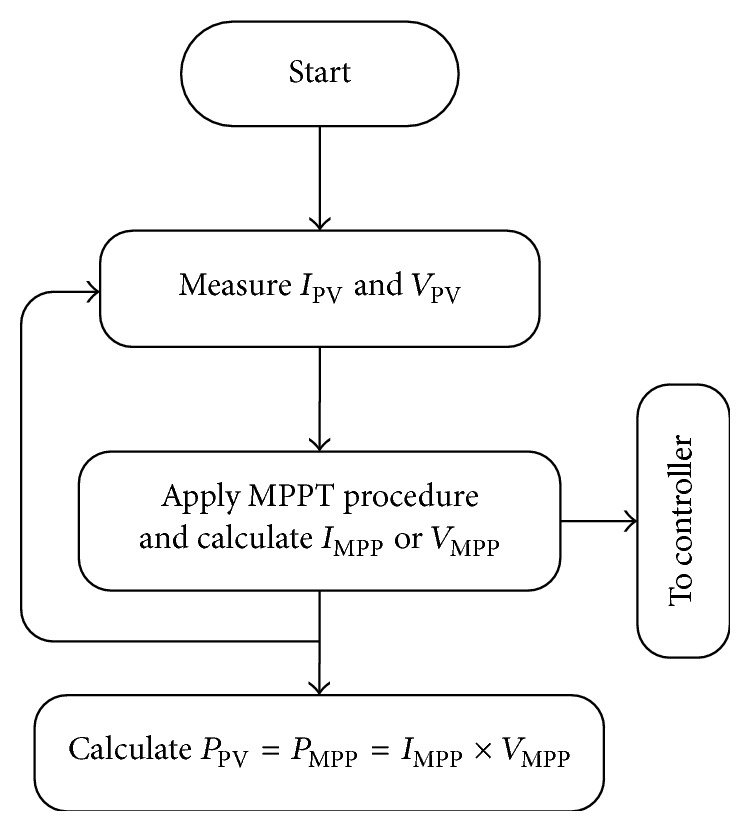
General MPPT algorithm.

Thus, in general, most of the MPPT algorithms usually start by sensing *I*_PV_ and *V*_PV_⁡, from the PV system terminals. Then the MPPT algorithm implements its own procedures (e.g., P&O) to find *I*_MPP_ or *V*_MPP_ to extract maximum power *P*_PV_ from the PV systems as shown in Figure 3, where *P*_PV_ is the product of *I*_MPP_ and *V*_MPP_. It should be noted that the MPPT algorithm only provides a reference to the controller of the DC-DC converter of a PV system [5]. It does not directly generate the duty ratio required for the converter to produce maximum power.

## 3. Characteristics of the Studied PV System

**Figure 4:**
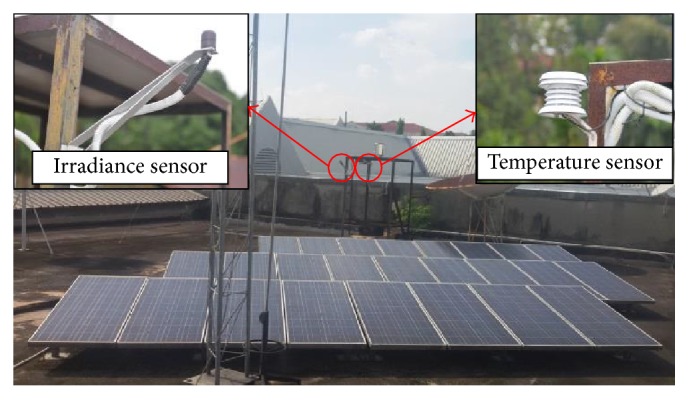
The studied 25 rooftop PV modules of SolarTIFSTF-120P6 PV with irradiance sensor and temperature sensor installed at the Universiti Kebangsaan Malaysia.

In this study, 25 SolarTIFSTF-120P6 PV modules are used with the capacity of 3 kW peak to supply the load, as shown in Figure 4. The modules are arranged in series-connected configuration, which produces a DC output voltage of 435 V. *G* and *T* are measured using a solar pyranometer sensor and a temperature sensor, respectively, as shown in Figure 4.

### 3.1. Sensors Characteristics

As mentioned earlier, two high-speed sensors are required to measure the irradiance and temperature. The irradiance sensor (S-LIB-M003) contains a silicon photodiode to measure solar power per unit area (W/m^2^). This silicon pyranometer smart sensor is designed to work with the HOBO® Weather Station Logger via its plug-in modular connector. In addition, all the calibration parameters are stored inside the S-LIB-M003 sensor, which automatically communicates configuration information to the logger without the need for any programming, calibration, or extensive setup.

Similarly, for temperature measurements, the smart sensor (S-TMB-M006) temperature is used. The stainless steel tip and a robust cable allow the S-TMB-M006 sensor to be immersed in water up to 50°C for 1 year. Thus, it is suitable for PV system condition monitoring. It can also automatically communicate configuration data information to the HOBO Weather Station without any programming, calibration, or extensive user setup. The silicon pyranometer smart sensor S-LIB-M003 uses the first channel and S-TMB-M006 smart sensor temperature uses the second channel out of 15 available channels of HOBO Weather Station.

### 3.2. PV Characteristics

**Figure 5:**
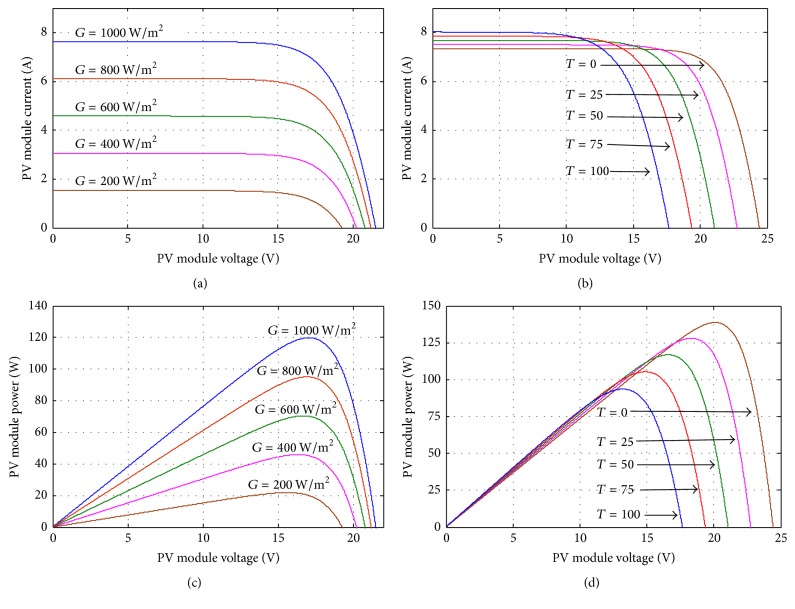
*I-V* and* P-V* characteristics of the SolarTIFSTF-120P6 PV panel: (a)* I-V* curves at *T* = 43.6°C, (b)* I-V* curves at *G* = 1000 W/m^2^, (c)* P-V* curves at *T* = 43.6°C, and (d)* P-V* curves at *G* = 1000 W/m^2^.

The characteristics of this PV module are depicted in Table 1, and the* I-V* and* P-V* curves obtained from (1) and (2), with varying irradiation and temperature values, for the SolarTIFSTF-120P6 module are exhibited in Figure 5. After a proper mathematical model is obtained, a suitable MPPT method is required to achieve better performance for the overall system. This is because the mathematical model cannot be directly used to generate reference currents due to computational burdens. Thus, in this study a new MPPT method is proposed as detailed in the succeeding section.

## 4. Proposed Random Forests MPPT Approach

**Figure 6:**
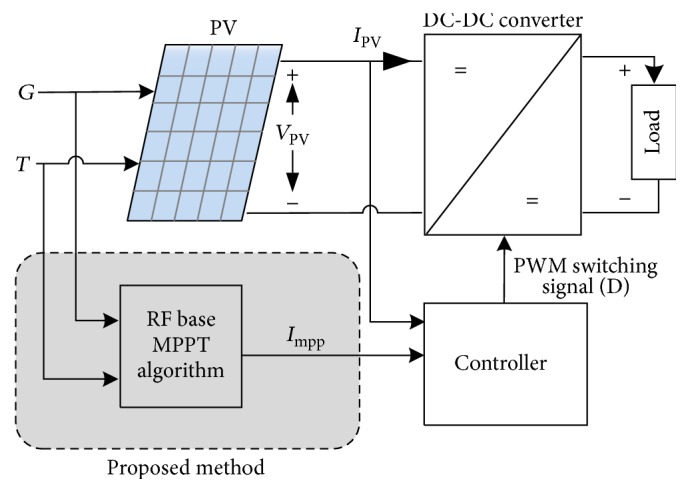
RF-based MPPT algorithm for controlling PV output power.

Unlike most of the MPPT algorithms such as IC, P&O, and HC, the proposed method uses *G* and *T* as inputs because these two measurements are commonly integrated in many modern PV systems for monitoring purposes as shown in Figure 4. Considering *G* and *T* are available as inputs, a recently developed RF soft computing approach is suggested to process the two inputs to generate the required reference current, *I*_MPP_, to the controller of the PV system as shown in Figure 6. Thus, this paper seeks development of a proper RF-based MPPT algorithm utilizing historical *G* and *T* data and target *I*_MPP_ values obtained from mathematical model described by (1). However, the designing of efficient control routine and the DC-DC converter is not the main focus of this work. An overview and adoption of RF to MPPT and in revaluation procedures are described in the following subsections.

### 4.1. Overview of Random Forests

**Figure 7:**
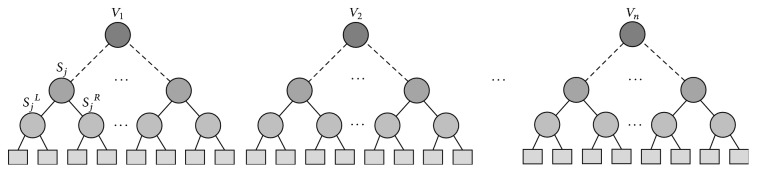
Random forests.

RF is an ensemble learning method for classification, regression, and other tasks, which operates by constructing a multitude of decision trees at training time and generating the class that is the mode of the classes (classification) or mean prediction (regression) of the individual trees. RF corrects the habit of decision trees in overfitting their training set. The training algorithm for RF applies the general technique of bootstrap aggregating or bagging to tree learners. The RF illustrated in Figure 7 classifies or predicts the value of a variable for an (*x*) input vector by building a number (*K*) of regression trees and averaging the results. After *K* and trees {*T*(*x*)}_1_^*K*^ are grown, the RF regression predictor is derived as

(3) $$\begin{array}{c} f_{\text{RF}}^{K}=\frac{1}{K}\overset{K}{\underset{n=1}{\sum }}T\left(\;x\;\right). \end{array}$$

In general, the RF algorithm for regression works as follows:

- *ntree* bootstrap samples *X*_*i*_ (*i* = bootstrap iteration) are randomly drawn with replacement from the original dataset, with each containing approximately one-third of the elements of the calibration dataset* X*. The elements not included in *X*_*i*_ are referred to as out-of-bag (OOB) data for the corresponding bootstrap sample.
- A regression tree for each of the bootstrap samples is grown (resulting in* ntree* trees) with the following modification: at each node, a subset of the predictor variables (*mtry*) is selected randomly to create the binary rule. In other words,* mtry* specifies the number of randomly chosen variables, upon which the decision for the best split at each node is made. Variable selection is based on the residual sum of squares; that is, the predictor with the lowest residual sum of squares is chosen for the split.* mtry* is held constant during the forest growing process.
- Each of the* ntree* trees is grown to the largest extent possible. No pruning is conducted.
- Lastly, predictions are calculated by placing each OOB observation or observation of the test data for each of the* ntree* trees. The predictions of all regression trees are then averaged to produce the final estimate [39].

The OOB error is an important feature of RF. As mentioned previously, each tree is built on a bootstrap sample that comprises roughly two-thirds of the training data. The remaining one-third (OOB) of the training data is not included in the learning sample for this tree and can be used in testing. Therefore, the RF model is applied to the OOB data. The deviations between predicted and reference values are then used to calculate the OOB error, which is the mean square error (MSE) for the regression. These OOB elements can be used by the* n*th tree to evaluate performance [40].

To avoid the correlation among the different trees, RF increases the diversity of the trees by making them grow from different bootstrap samples created by a procedure called bagging (bagging =* mtry* = number of predictors) [35]. This procedure increases generality, makes the regression more robust at slight variations in the training data, and generally increases overall prediction accuracy [39]. When RF reflects the growth of a tree, the best split based on a number of randomly sampled predictor variables is used. If all variables were used for each tree, the trees would become identical and therefore highly correlated [39]. Thus, the randomly chosen subsets of predictor variables at each split of each tree ensure lower correlation between trees, which in turn increases model robustness.

RF is efficient to apply for MPPT prediction for PV systems because it involves a combination of robust characteristics. The approach does not require the specification of an underlying PV system model, and it offers the ability to capture nonlinear association of patterns between predictors, such as irradiance and temperature, to determine accurate reference current (*I*_MPP_) and calculate MPP. The approach is also able to handle highly correlated predictor variables. Moreover, the approach offers the flexibility to perform a number of statistical data analyses and is computationally lighter than other tree ensemble methods [33, 39, 41].

### 4.2. Random Forests Training

In this study, a RF-based MPPT system is considered for the 3 kW PV system, with the use of SolarTIFSTF-120P6 PV modules described in Section 2. The input data samples that correspond to irradiance (*G*) and temperature (*T*) are generated using (4) and (5), respectively, and the derived values are used to obtain the target data for training using the parameters given in Table 1 and (1).

(4) Gi=rand∗Gmax−Gmin+Gmin,

(5) Ti=rand∗Tmax−Tmin+Tmin,

where* i* is the number of data samples, from 1 to number of data samples (*m*).

The minimum and maximum limits for the *G* and *T* are selected based on historical data. In Malaysia, *G* typically varies between 0 W/m^2^ and 1200 W/m^2^, while *T* fluctuates from 20°C to 35°C [42]. Therefore, *G*_min_, *G*_max_, *T*_min_, and *T*_max_ in (4) and (5) are identified as 0 W/m^2^, 1500 W/m^2^, 0°C, and 45°C, respectively, to ensure that the generated data cover the typical and historical data range. Since the studied system is small, effect of partial shading on some modules of the system is not considered in this work in tracking MPPT.  Table 2 shows some of the data samples which are used for training.

In general, two parameters must be adjusted in RF: the overall number of trees in a forest (*ntree*) and the number of data samples (*m*). The tuning is based on the performance of OOB data. An important consideration is to determine how many trees should be grown according to the RF model. Breiman [39] suggested that the generalization error converges as the number of trees increases. Adding an increasing number of trees to the model does not result in overadjustment. The main limitation of increasing* ntree* is the additional computation time.

**Figure 8:**
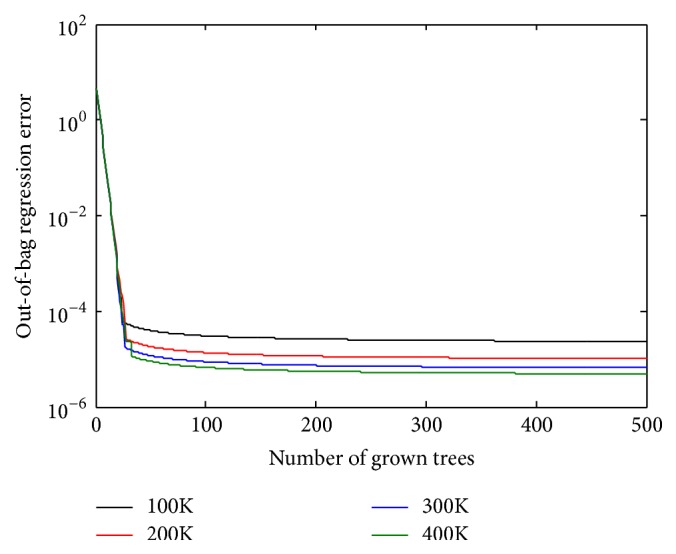
Training error rates based on the RF-based MPPT.

**Figure 9:**
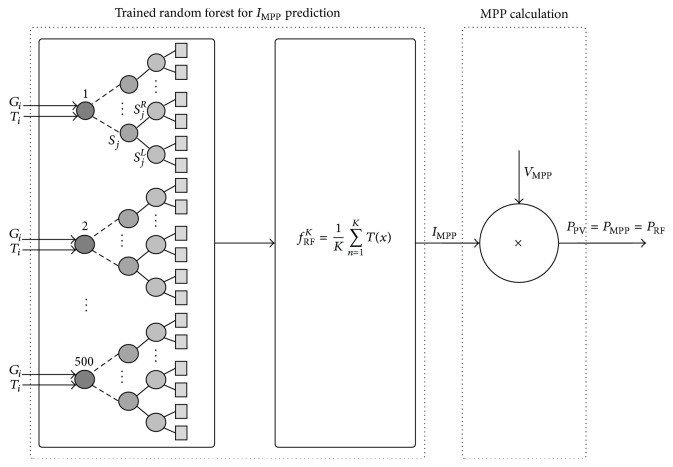
Block diagram of the trained RF-based MPPT algorithm with external MPP calculation.

To assess the optimal value of* ntree* and the optimal value of the number of data samples (*m*), four RF models are created using 500 trees with different numbers of data samples. The MSE values are then averaged.  Figure 8 shows how the error rates (2.582*E* − 5, 1.148*E* − 5, 7.38*E* − 6, and 5.60*E* − 6) change with the number of trees, when data samples used are equal to 100,000, 200,000, 300,000, and 400,000, respectively. From approximately 200 trees, the MSE of each dataset stabilizes, and increasing the number of trees neither increases nor decreases the MSE. Therefore, 200 trees in the RF can be regarded as sufficient. As shown in Figure 8, the values for MSE for the 300,000 and 400,000 data samples slightly differ. Hence, 300,000 data samples are used because increasing the data samples beyond 300,000 leads to an increase in computation time, which will not be beneficial. After the RF is trained, the approach can be used to generate reference current, *I*_MPP_, and calculate MPP with new input data as shown in Figure 9. It should be noted from Figure 9 that the trained RF only estimate the reference current, *I*_MPP_, and MPP is then calculated simply by multiplying *I*_MPP_ and *V*_MPP_, where *V*_MPP_ is the system voltage at *I*_MPP_ as shown in Figure 2.

### 4.3. Performance Evaluation

The main concern in forecasting is to test the performance of the developed forecasting technique for its suitability and accuracy. For this purpose, the Bland–Altman test is conducted first. The Bland–Altman test is a type of statistical analysis typically used to compare measured values and a reference value. If the differences between the RF-based MPP and the reference MPP are within the *µ* ± 2*σ* (95%) limits of acceptability, the proposed method is considered as an accurate model [43–45]. In the above expression, *µ* is the mean difference (bias) of the power measurements between the proposed system and the reference and *σ* is the standard deviation for the difference of the power measurements.

To evaluate the performance of the various MPPT methods, namely, ANFIS, ANN, and the proposed RF-based method, three standard error measurements are used: mean error (ME), mean square error (MSE), and standard deviation of the error (*σ*). These indices are given by [33]

(6) ME=1N∑i=1NPmathi−PRFi,MSE=1N∑i=1NPmathi−PRFi2,σ=1N∑i=1NPmathi−µ2,

where *P*_RF*i*_ is the* i*th power measured using RF, *P*_math*i*_ is the *i*th reference power based mathematical model, and *µ* is the average of the measured values.

## 5. Results and Discussion

To validate the MPPT algorithms, the developed and trained RF-based MPPT is verified using the actual SolarTIFSTF-120P6 PV modules output data instead of testing based on the slow change of metrological conditions (ramp) or step change implemented in previous research. The data were obtained from a 3 kW rooftop PV system at Universiti Kebangsaan Malaysia from March 1, 2013, to February 15, 2014, using a high sampling data logger at a sampling rate of 30 seconds. *G* and *T* are measured using a solar pyranometer sensor S-LIB-M003 and a temperature sensor S-TMB-M006, respectively, as explained in Section 3.

**Figure 10:**
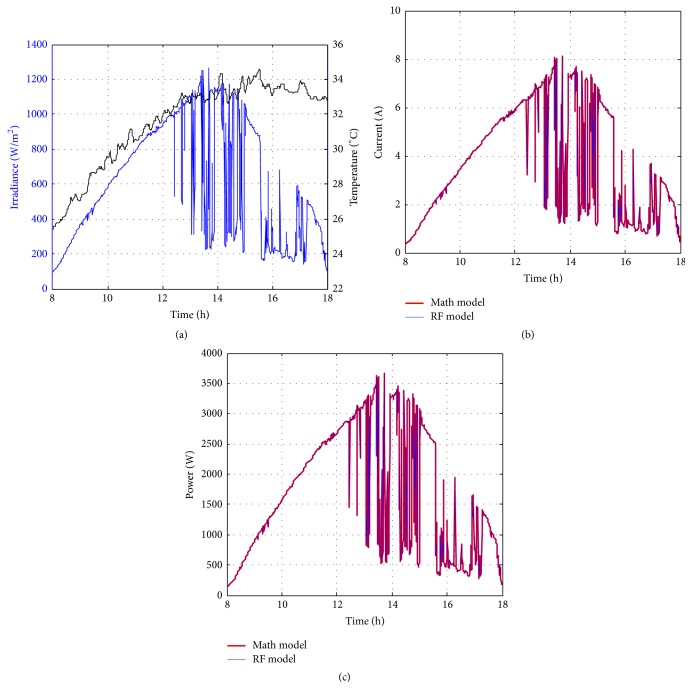
Performance of RF-based MPPT method: (a) metrological conditions, (b)* estimated I*_MPP_, and (c) extracted maximum electrical power on March 1, 2013.

**Figure 11:**
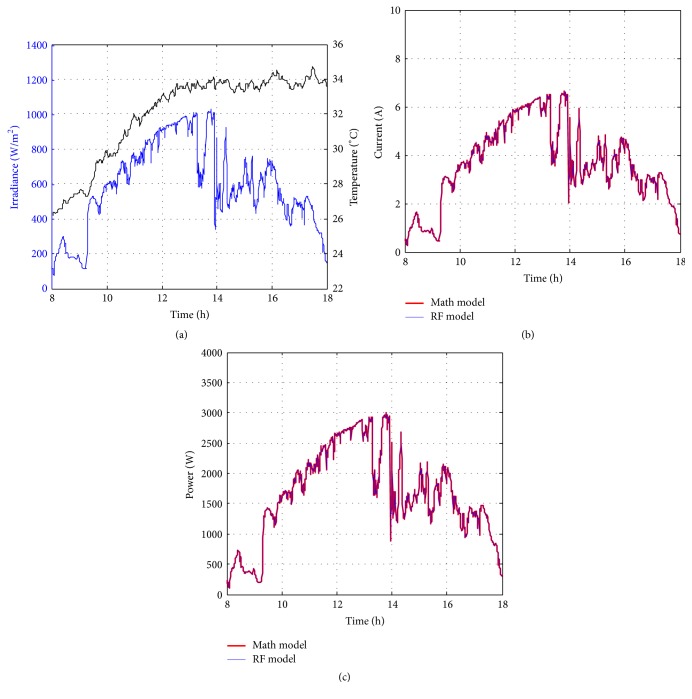
Performance of RF-based MPPT method: (a) metrological conditions, (b)* estimated I*_MPP_, and (c) extracted maximum electrical power on June 1, 2013.

**Figure 12:**
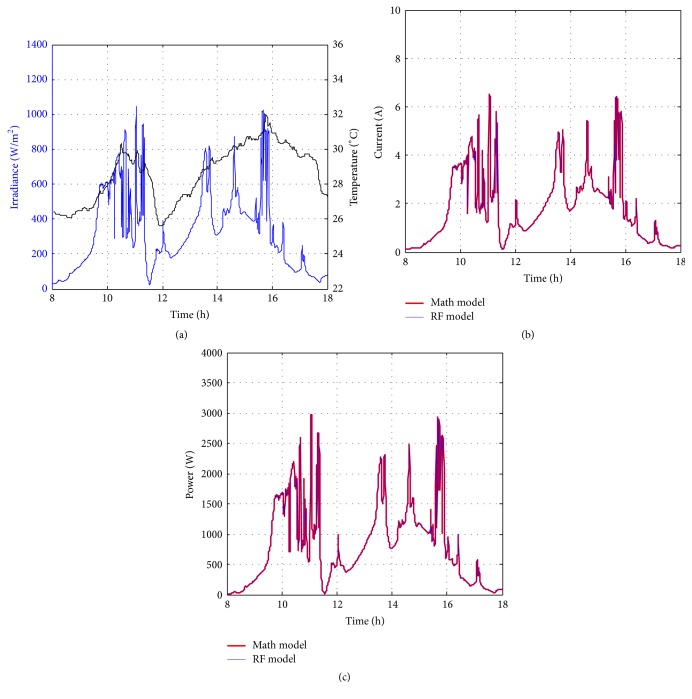
Performance of RF-based MPPT method: (a) metrological conditions, (b)* estimated I*_MPP_, and (c) extracted maximum electrical power on July 1, 2013.

**Figure 13:**
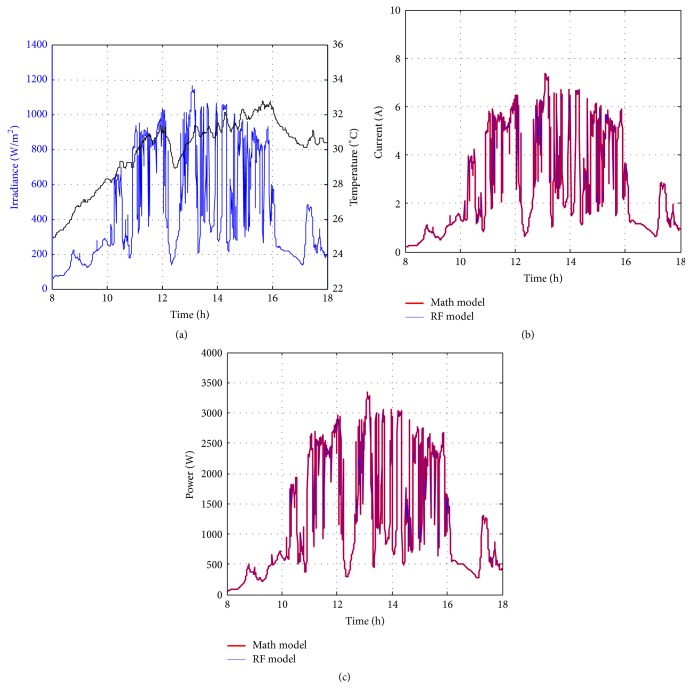
Performance of RF-based MPPT method: (a) metrological conditions, (b)* estimated I*_MPP_, and (c) extracted maximum electrical power on August 15, 2013.

**Figure 14:**
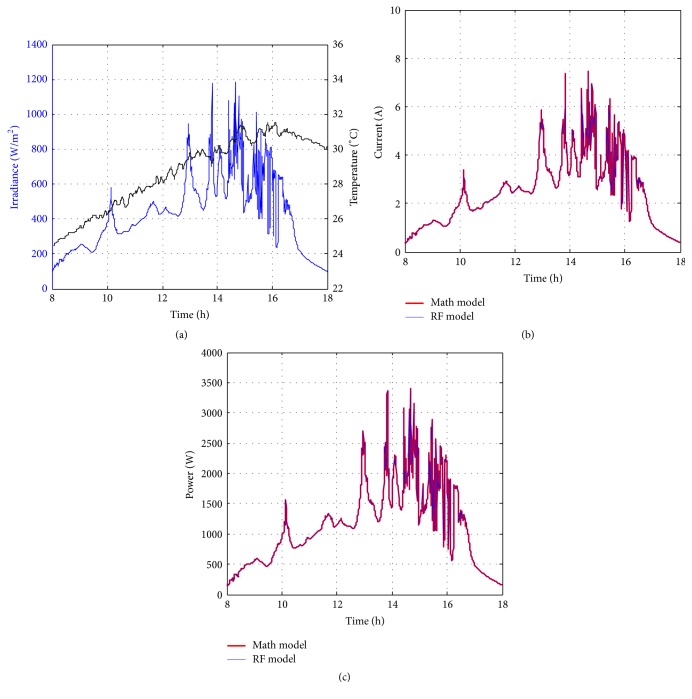
Performance of RF-based MPPT method: (a) metrological conditions, (b)* estimated I*_MPP_, and (c) extracted maximum electrical power on October 15, 2013.

**Figure 15:**
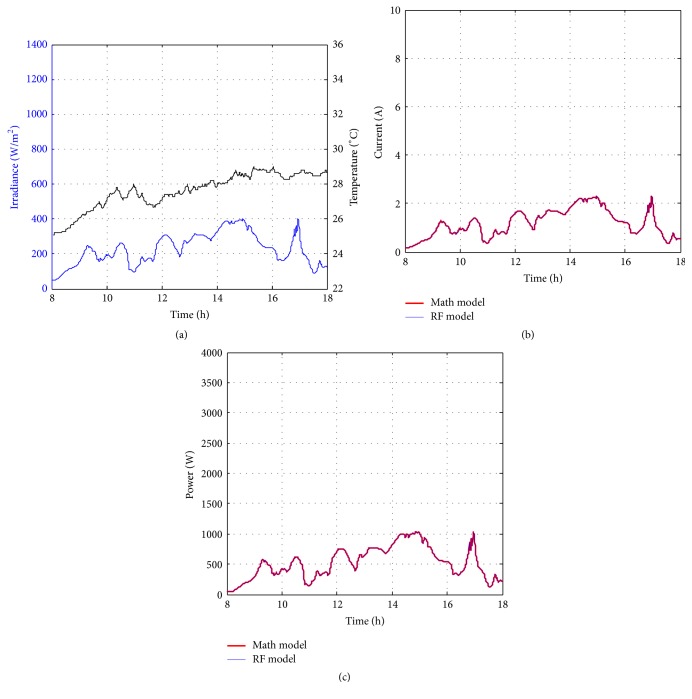
Performance of RF-based MPPT method: (a) metrological conditions, (b)* estimated I*_MPP_, and (c) extracted maximum electrical power on December 1, 2013.

**Figure 16:**
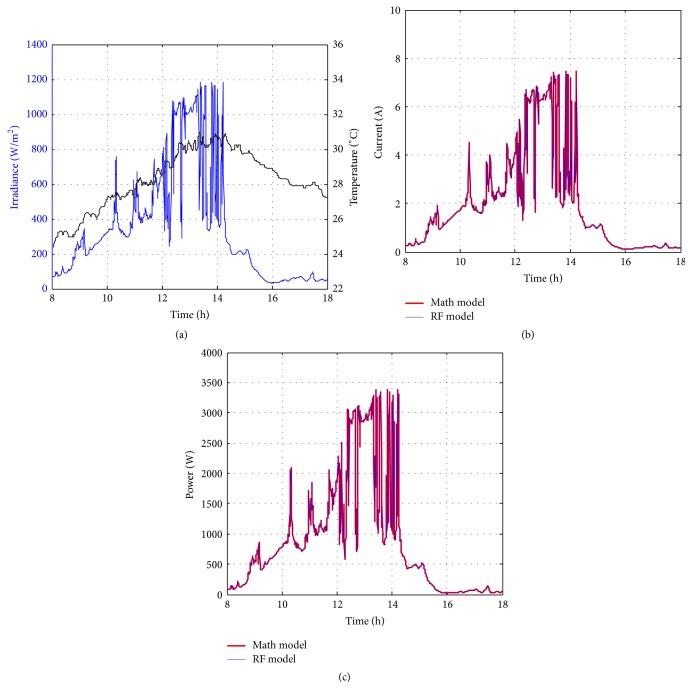
Performance of RF-based MPPT method: (a) metrological conditions, (b)* estimated I*_MPP_, and (c) extracted maximum electrical power on January 15, 2014.

**Figure 17:**
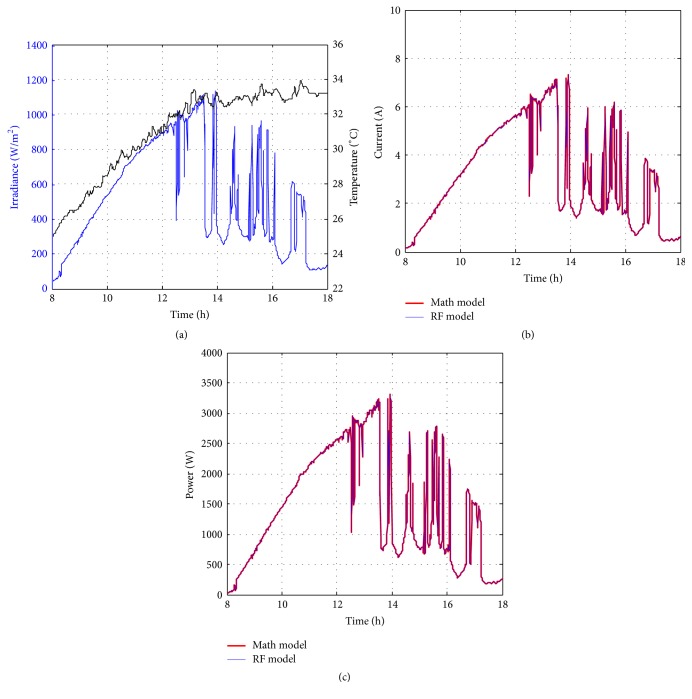
Performance of RF-based MPPT method: (a) metrological conditions, (b)* estimated I*_MPP_, and (c) extracted maximum electrical power on February 15, 2014.

Figures 10–17 show the meteorological operation conditions (*G* and *T*) and the output maximum electrical power (*P*_PV_ = *P*_MPP_ = *V*_MPP_*∗I*_MPP_) extracted from the developed RF-based MPPT (RF model) for selected days that have varying patterns. From these figures, the output power of the system is shown to be highly dependent on *G* and follows the same pattern.

**Figure 18:**
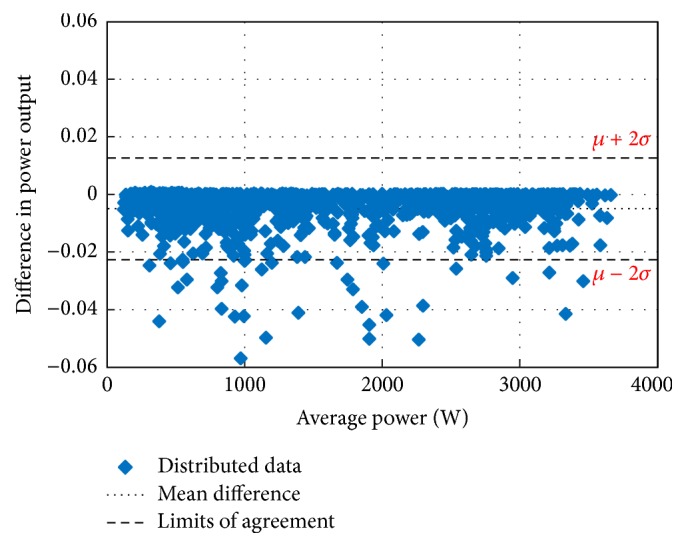
Bland–Altman test for power measurements on March 1, 2013.

**Figure 19:**
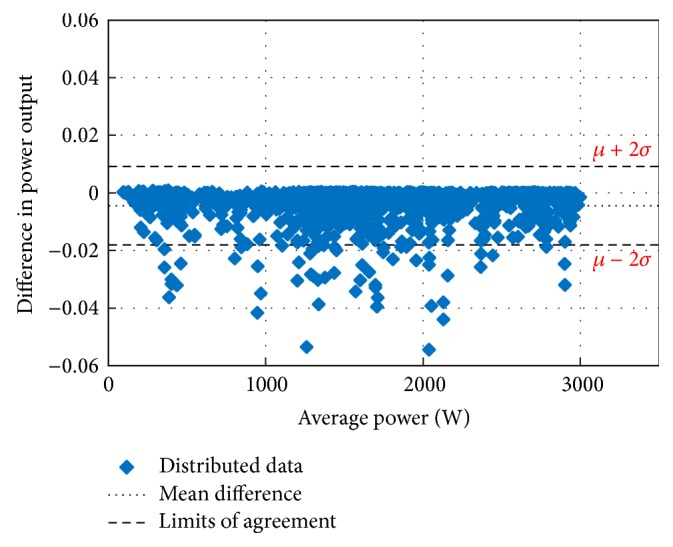
Bland–Altman test for power measurements on June 1, 2013.

**Figure 20:**
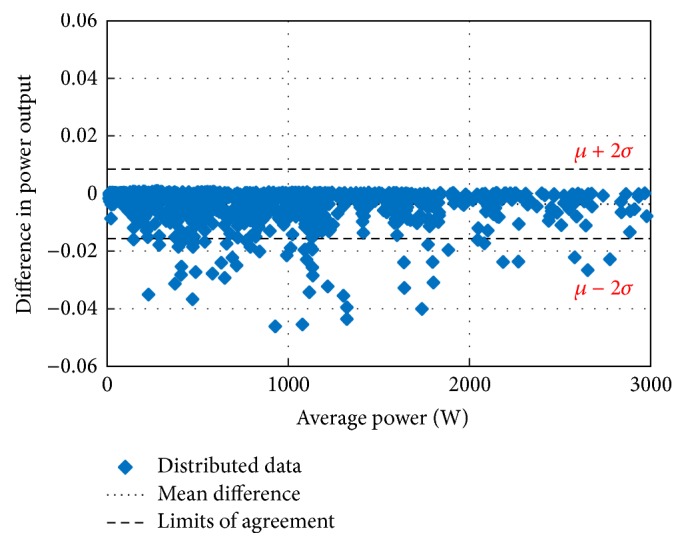
Bland–Altman test for power measurements on July 1, 2013.

**Figure 21:**
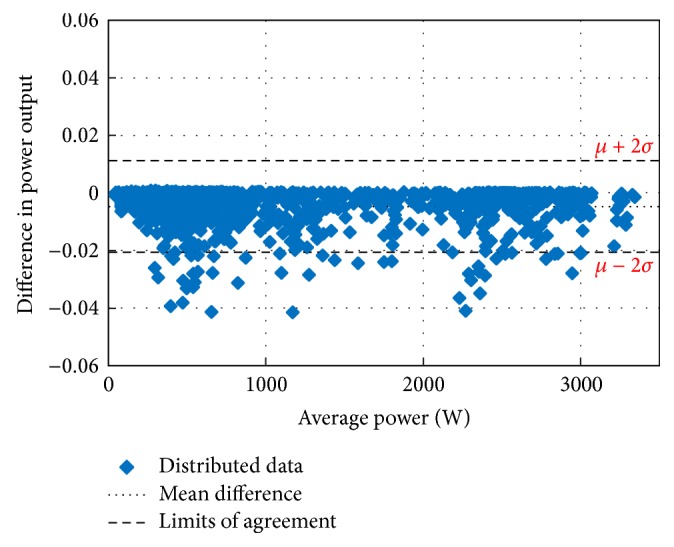
Bland–Altman test for power measurements on August 15, 2013.

**Figure 22:**
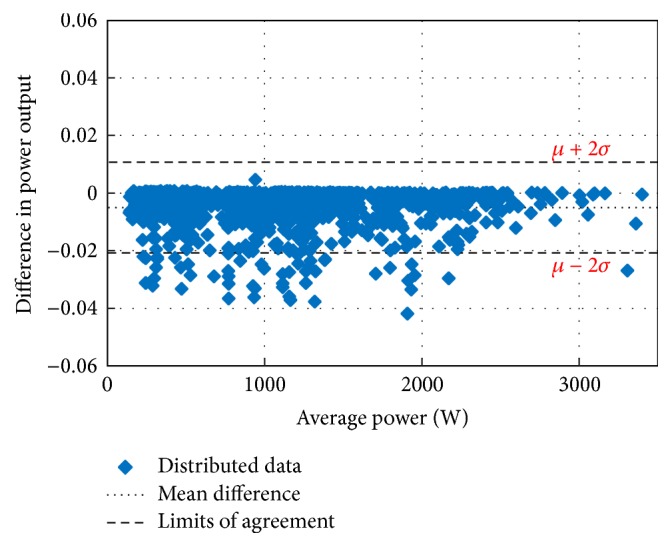
Bland–Altman test for power measurements on October 15, 2013.

**Figure 23:**
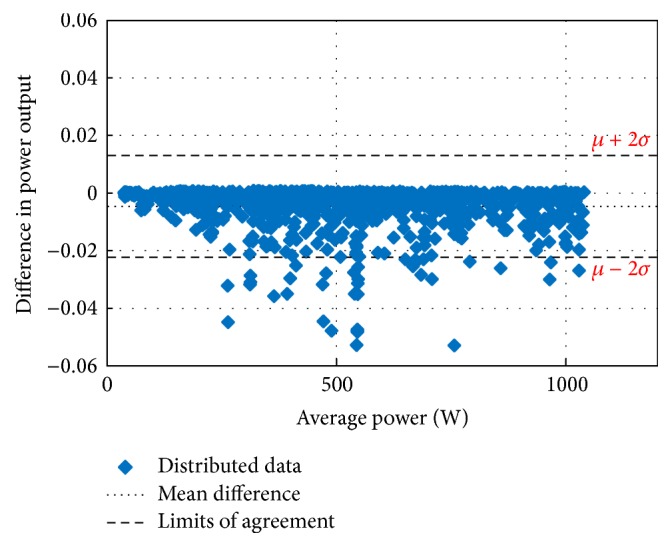
Bland–Altman test for power measurements on December 1, 2013.

**Figure 24:**
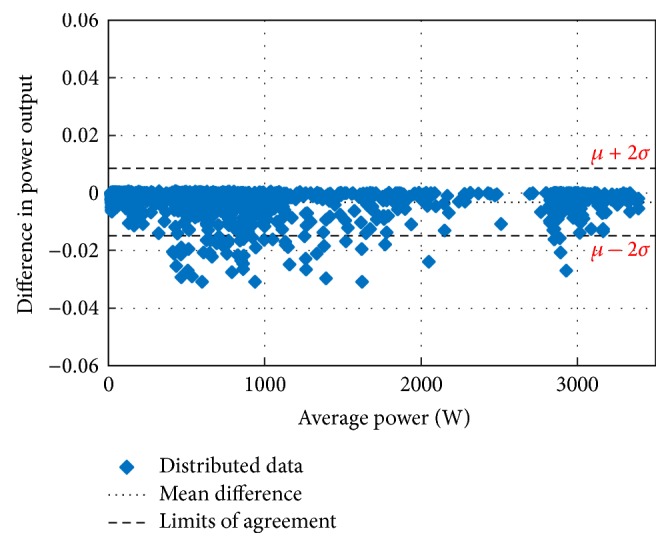
Bland–Altman test for power measurements on January 15, 2014.

**Figure 25:**
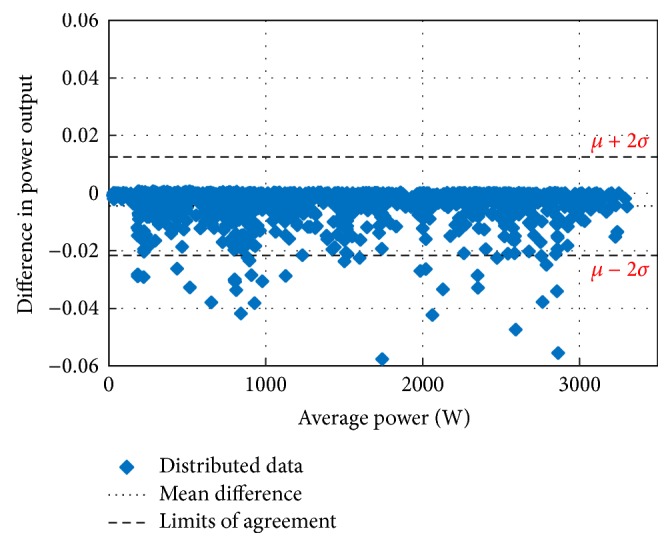
Bland–Altman test for power measurements on February 15, 2014.

 Figures 10–17 do not clearly show how close the measured MPP based RF (RF model) is from the reference MPP based mathematical model (math model) which have been obtained using the data given in Table 1 and (1). Therefore, to show the difference or symmetry between the powers measured using RF-based MPPT (RF model) and the reference power based mathematical model (math model), the Bland–Altman test is conducted. For the selected days, the corresponding Bland–Altman test plots are exhibited in Figures 18–25. In these figures, a regression line is added to the plots as a dotted line to show the limit of acceptability between the proposed MPP and the reference MPP. From the figures, most of the data fall within the *µ* ± 2*σ* (95%) limits of acceptability [43–45]. This observation indicates that the proposed RF-based MPPT is accurate and effective.

To show the reliability of the proposed MPPT algorithm, tests were conducted for an entire year. Measurement data and results of the Bland–Altman test conducted at the beginning and at the end of each month are shown in Table 3. As shown in this table, most of the measured data from the proposed MPP tracker and the reference power lie between 95% and 96.83% limits of acceptability. These statistical results support and promote the validity of the measurement of the power of the proposed MPP tracker relative to the reference power.

### 5.1. Performance Comparison

For a fair comparison and to show the superiority of the RF-based MPPT method, the new MPPT methods are compared with well-known approaches, namely, ANN and ANFIS-based MPPT methods. For the appraisal of the techniques, three indices mentioned in Section 4.3 are calculated.

The first indices, where mean error (ME) is calculated for the proposed RF model, ANN, and ANFIS for 24 days from March 1, 2013, to February 15, 2014, are shown in Table 4. The best performance is boldfaced, which clearly shows that the RF approach provides better results with very low ME values than those of ANN and ANFIS models. For RF-based MPPT, the best ME result obtained was 0.002985 on January 15, 2014, and the worst result recorded was 0.005087 on May 1, 2013. In the 24 days, the average ME values for RF, ANN, and ANFIS were 0.00439, 0.03083, and 0.89843, respectively. These values clearly indicate that the proposed method outperformed the other methods in terms of ME.

The second index compares the performances of MSE of various MPPT methods. MSE is inversely proportional to the quality of the signal. A decrease in MSE value means an increase in the quality of the signal. In other words, a decrease in the MSE value means the output is closer to the reference MPP, whereas an increase in the MSE value implies that the output is spread out from the true MPP. The performance of RF, ANN, and ANFIS-based MPPT methods in terms of MSE is listed in Table 5. The MSE value for the RF model decreases from 3.445*E* − 05 on January 15, 2014, to 0.000109 on May 1, 2013. In the 24 days, the average MSE values for RF, ANN, and ANFIS were 7.432*E* − 05, 0.004893, and 4.689996, respectively. The results again show that the RF-based MPPT achieves better results than the other techniques.

The third index compares the standard deviations, *σ*, of the various MPPT methods. *σ* evaluates the rate of variation against the average value. A low standard deviation indicates that the data points are near the mean, while a greater *σ* indicates that the data points are scattered in a wide surrounding range from the mean. The calculated standard deviations of the RF model, ANN, and ANFIS for 24 days, from March 1, 2013, to February 15, 2014, are depicted in Table 6. Table 6 clearly shows that the RF-based MPPT performs better than the other methods at varying *σ* between 0.005054 on January 15, 2014, and 0.009126 on May 1, 2013. In the 24 days, the average *σ* values for RF, ANN, and ANFIS were 0.007295, 0.059747, and 1.878943, respectively.

## 6. Conclusion

This paper introduced a new and effective MPPT algorithm based on RF for a 3 kW peak PV system composed of 25 SolarTIFSTF-120P6 PV modules. With the bootstrapping method used in the training procedures and proper parameter selection of the random forests, better MPPT model performance was achieved. To evaluate the reliability and efficiency of the proposed algorithm, the RF model was tested using actual data obtained from March 1, 2013, to February 15, 2014, every 15th of the month, and the performance of the proposed RF technique was compared with that of ANN and ANFIS methods. The performance of the proposed RF model was evaluated based on the Bland–Altman test results and on the obtained ME, MSE, and *σ* values. The results showed that the RF-based MPPT passed the Bland–Altman test with more than 95% limits of acceptability in all tested cases. Furthermore, comparative analysis reveals that the proposed MPPT method outperforms both ANN and ANFIS algorithms in terms of ME, MSE, and *σ* by a significant margin when tested under the same strict meteorological and technical conditions. Finally, the proposed method is found to respond quickly to fast-changing environmental conditions; thus the method can be adopted for real-time MPPT. The extension of this work is under way to develop a DC-to-DC boost converter hardware based on the proposed MPPT algorithm.

## Figures and Tables

**Table 1:** PV module characteristics.

PV module type: SolarTIFSTF-120P6	
Number of modules in series	25
Number of modules in parallel	1
Maximum power (*P*_MPP_)	3 kW
Open circuit voltage (*V*_oc_)	537.5 V
Short circuit current (*I*_sc_)	7.63 A
Voltage at maximum power (*V*_MPP_)	435 V
Current at maximum power (*I*_MPP_)	6.89 A
Current temperature coefficient (*α*)	6.928 mA/°C
Voltage temperature coefficient (*β*)	−0.068 V/°C

**Table 2:** Training data samples.

Sample number	*G* from (4)	*T* from (5)	*I*_MPP_ from (1)
1	426.988	11.170	2.103
2	654.855	43.278	4.405
3	624.986	41.217	4.128
4	51.2322	7.3070	0.170
5	726.523	33.586	4.577
6	657.228	22.346	3.753
7	821.156	25.784	4.899
8	332.130	19.744	1.712
9	191.898	21.386	0.891
10	828.676	33.751	5.268
⋮	⋮	⋮	⋮
399990	1043.98	1.6760	5.072
399991	228.114	1.0788	0.885
399992	623.211	11.358	3.207
399993	606.037	28.896	3.631
399994	225.997	24.816	1.133
399995	98.2440	42.387	0.437
399996	241.430	3.9415	0.985
399997	972.252	9.7257	5.089
399998	538.437	25.532	3.105
399999	673.546	15.412	3.624
400000	700.615	7.5407	3.510

**Table 3:** Bland–Altman test.

Number	Date	*µ*	*µ* + 2*σ*	*µ* − 2*σ*	Bland–Altman (limits of agreement%)
1	March 1, 2013	−0.004958	0.012704	−0.022622	96.33
2	March 15, 2013	−0.005070	0.010587	−0.020727	95.00
3	April 1, 2013	−0.004392	0.010761	−0.019546	96.17
4	April 15, 2013	−0.004566	0.009402	−0.018535	95.67
5	May 1, 2013	−0.005087	0.013169	−0.023345	95.83
6	May 15, 2013	−0.003066	0.008015	−0.014149	95.33
7	June 1, 2013	−0.004477	0.009100	−0.018054	95.42
8	June 15, 2013	−0.005004	0.012168	−0.022177	96.08
9	July 1, 2013	−0.003603	0.008422	−0.015630	95.58
10	July 15, 2013	−0.004724	0.011462	−0.020911	96.33
11	August 1, 2013	−0.003698	0.009993	−0.017389	96.25
12	August 15, 2013	−0.004407	0.008342	−0.017157	96.08
13	September 1, 2013	−0.004319	0.012757	−0.021397	96.67
14	September 15, 2013	−0.003915	0.009265	−0.017096	95.42
15	October 1, 2013	−0.004564	0.008303	−0.017431	95.33
16	October 15, 2013	−0.004691	0.008997	−0.018379	95.83
17	November 1, 2013	−0.004458	0.009226	−0.018142	95.17
18	November 15, 2013	−0.004125	0.008517	−0.016768	95.83
19	December 1, 2013	−0.004573	0.013109	−0.022256	96.33
20	December 15, 2013	−0.004813	0.010291	−0.019918	95.08
21	January 1, 2014	−0.004755	0.011843	−0.021353	95.92
22	January 15, 2014	−0.002985	0.007125	−0.013096	95.42
23	February 1, 2014	−0.004661	0.009694	−0.019016	95.08
24	February 15, 2014	-0.004487	0.012655	-0.021630	96.83

**Table 4:** Mean error.

Number	Date	RF	ANN	ANFIS
1	March 1, 2013	0.004958	0.016018	0.463951
2	March 15, 2013	0.005070	0.019042	0.664193
3	April 1, 2013	0.004392	0.028661	0.574925
4	April 15, 2013	0.004566	0.009100	0.254380
5	May 1, 2013	0.005087	0.023952	0.796137
6	May 15, 2013	0.003066	0.048598	1.494458
7	June 1, 2013	0.004477	0.013066	0.310572
8	June 15, 2013	0.005004	0.033858	0.872664
9	July 1, 2013	0.003603	0.049649	1.280312
10	July 15, 2013	0.004724	0.030334	1.215951
11	August 1, 2013	0.003698	0.057225	1.321636
12	August 15, 2013	0.004407	0.026956	0.828955
13	September 1, 2013	0.004319	0.042546	1.334692
14	September 15, 2013	0.003915	0.040017	1.592547
15	October 1, 2013	0.004564	0.041882	1.311885
16	October 15, 2013	0.004691	0.020095	0.804006
17	November 1, 2013	0.004458	0.024107	0.541576
18	November 15, 2013	0.004125	0.032625	0.805526
19	December 1, 2013	0.004573	0.052986	1.609900
20	December 15, 2013	0.004813	0.020999	0.680179
21	January 1, 2014	0.004755	0.026556	0.843890
22	January 15, 2014	0.002985	0.052114	0.929768
23	February 1, 2014	0.004661	0.010587	0.264384
24	February 15, 2014	0.004487	0.018884	0.765933

Average		0.004392	0.030827	0.898434

**Table 5:** Mean square error.

Number	Date	RF	ANN	ANFIS
1	March 1, 2013	0.000102	0.001797	2.049067
2	March 15, 2013	8.694**E** − 05	0.002061	3.576209
3	April 1, 2013	7.665**E** − 05	0.005982	2.722844
4	April 15, 2013	6.959**E** − 05	0.001079	1.132424
5	May 1, 2013	0.000109	0.002648	4.146236
6	May 15, 2013	4.008**E** − 05	0.007295	6.589908
7	June 1, 2013	6.609**E** − 05	0.001242	1.738047
8	June 15, 2013	9.871**E** − 05	0.003777	4.251256
9	July 1, 2013	4.911**E** − 05	0.008876	6.470000
10	July 15, 2013	8.777**E** − 05	0.004461	7.752110
11	August 1, 2013	6.050**E** − 05	0.012030	7.113411
12	August 15, 2013	6.003**E** − 05	0.003355	3.285035
13	September 1, 2013	9.151**E** − 05	0.008465	7.505080
14	September 15, 2013	5.872**E** − 05	0.007030	9.549102
15	October 1, 2013	6.218**E** − 05	0.007597	7.358378
16	October 15, 2013	6.881**E** − 05	0.002194	3.970052
17	November 1, 2013	6.665**E** − 05	0.002759	2.345395
18	November 15, 2013	5.694**E** − 05	0.006170	4.068883
19	December 1, 2013	9.902**E** − 05	0.008211	8.435125
20	December 15, 2013	8.016**E** − 05	0.003226	3.616879
21	January 1, 2014	9.143**E** − 05	0.004822	4.820838
22	January 15, 2014	3.445**E** − 05	0.009134	4.257238
23	February 1, 2014	7.320**E** − 05	0.001236	1.057766
24	February 15, 2014	9.358**E** − 05	0.001975	4.748613

Average		7.432**E** − 05	0.004893	4.689996

**Table 6:** Standard deviation.

Number	Date	RF	ANN	ANFIS
1	March 1, 2013	**0.008829**	0.039252	1.354749
2	March 15, 2013	**0.007826**	0.041222	1.770712
3	April 1, 2013	**0.007574**	0.071872	1.546796
4	April 15, 2013	**0.006982**	0.031564	1.033328
5	May 1, 2013	**0.009126**	0.045551	1.874281
6	May 15, 2013	**0.005539**	0.070252	2.087669
7	June 1, 2013	**0.006787**	0.032738	1.281277
8	June 15, 2013	**0.008584**	0.051308	1.868246
9	July 1, 2013	**0.006011**	0.080083	2.198218
10	July 15, 2013	**0.008091**	0.059517	2.504955
11	August 1, 2013	**0.006843**	0.093587	2.316925
12	August 15, 2013	**0.006373**	0.051277	1.611967
13	September 1, 2013	**0.008536**	0.081591	2.392730
14	September 15, 2013	**0.006588**	0.073689	2.648585
15	October 1, 2013	**0.006432**	0.076453	2.374608
16	October 15, 2013	**0.006840**	0.046676	1.432597
17	November 1, 2013	**0.006320**	0.071462	1.849473
18	November 15, 2013	**0.006320**	0.071462	1.849473
19	December 1, 2013	**0.008838**	0.073528	2.417747
20	December 15, 2013	**0.007550**	0.052777	1.776125
21	January 1, 2014	**0.008296**	0.064166	2.027135
22	January 15, 2014	**0.005054**	0.080129	1.842142
23	February 1, 2014	**0.007175**	0.033526	0.993944
24	February 15, 2014	**0.008571**	0.040242	2.040938

Average		**0.007295**	0.059747	1.878943
